# The roles of rat medial prefrontal and orbitofrontal cortices in relapse to cocaine-seeking: A comparison across methods for identifying neurocircuits

**DOI:** 10.1016/j.addicn.2022.100031

**Published:** 2022-07-28

**Authors:** Javier R. Mesa, Daniel W. Wesson, Marek Schwendt, Lori A. Knackstedt

**Affiliations:** a Department of Psychology, University of Florida, 114 Psychology, 945 Center Dr., Gainesville, FL 32611, USA; b Department of Pharmacology and Therapeutics, University of Florida, Gainesville, FL, USA; c Center for Addiction Research and Education, University of Florida, Gainesville, FL, USA

**Keywords:** Prelimbic, Infralimbic, Orbitofrontal, Cocaine, Reinstatement, Relapse

## Abstract

A large body of research supports the notion that regions of the rodent frontal cortex regulate reinstatement of cocaine seeking after cessation of intravenous cocaine self-administration. However, earlier studies identifying the roles of medial (mPFC) and orbital prefrontal cortices (OFC) in reinstatement relied on pharmacological inactivation methods, which indiscriminately inhibited cells within a target region. Here, we first review the anatomical borders and pathways of the rat mPFC and OFC. Next, we compare and contrast findings from more recent cocaine seeking and reinstatement studies that used chemogenetics, optogenetics, or advanced tracing to manipulate specific local cell types or input/output projections of the mPFC and OFC subregions. We found that these studies largely corroborated the roles for mPFC subregions as ascribed by pharmacological inactivation studies. Namely, the prelimbic cortex generally drives cocaine seeking behaviors while the infralimbic cortex is recruited to inhibit cocaine seeking by extinction training but may contribute to seeking after prolonged abstinence. While the OFC remains understudied, we suggest it should not be overlooked, and, as with prelimbic and infralimbic cortices, we identify specific pathways of interest for future studies.

## 1. Introduction

Cocaine use disorder (CUD) is a chronic condition marked by a high risk for relapse, even after years of abstinence. In 2020, approximately 1.3 million Americans had a diagnosis of CUD [1]. There are currently no FDA-approved pharmacotherapies for CUD or cocaine craving. CUD is associated with adaptations across many cortical and subcortical brain regions, though the altered function and/or structure of the prefrontal cortex (PFC) in CUD is one of the most reproduced findings. The medial PFC (mPFC) is known to modulate learned behaviors and both inhibition and stimulation of goal-driven behaviors [2] and is comprised of the anterior cingulate (ACC), prelimbic (PL), and infralimbic cortices (IL). Within the context of substance use disorders, the mPFC plays a key role in cocaine seeking and relapse [2, 3]. The orbitofrontal cortex (OFC) is critical for cognitive flexibility in response to changes in the predictive valuation of cocaine-associated cues and contexts. As cocaine seeking necessitates goal-directed action in response to drug-associated cues and changes in drug reinforcement, both the mPFC and OFC are potential targets for interventions to prevent relapse. Recent advances in the technologies used to manipulate brain regions and circuits in preclinical animal models of cocaine-seeking have helped elucidate the functionality and roles of frontal cortical regions in cocaine seeking.

With an emphasis on rat models of relapse to cocaine seeking and such new technologies, this review aims to: (1) summarize earlier neuroanatomical literature defining the mPFC and OFC and their projection networks; (2) summarize findings from pharmacological inactivation, chemogenetic, optogenetic, and advanced tracing studies regarding the roles of principal mPFC and OFC subregions and their projections in mediating cocaine reinstatement; (3) compare results from classical pharmacological inactivation and chemo-/optogenetic studies; and (4) propose new directions to better understand the roles of the mPFC and OFC in reinstatement. We note that all studies reviewed here used only male rats.

## 2. Anatomy of the orbital and medial PFC

### 2.1. Anatomical boundaries of the mPFC

The mPFC has been historically difficult to distinguish using only anatomical criteria. Brodmann’s characterizations of the primate frontal area (1909) were based on cytoarchitectonics and identified a developed granular frontal cortex in primates relative to other mammals. This feature was thought to be uniquely primate and future researchers would equate Brodmann’s granular frontal cortex with the whole of what now many consider as prefrontal cortex. But Brodmann’s granular frontal cortex excluded the non-motor anterior cingulate region (Brodmann’s areas 24, 25, and 32) along the medial wall that lacked a granular layer [4, 5]. Despite this distinction, both the granular frontal and anterior cingulate regions were commonly designated as “prefrontal.” Brodmann’s exceptionalist framework was also disputed because it conflicted with notions of a common organization of brain structures across mammals and other means of characterizing PFC emerged [4]. Rose and Woolsey [6] proposed defining primate PFC based on extensive reciprocal projections with the mediodorsal thalamus (MDT) and similar projections were later identified in rats [5, 7]. Although this anatomical criterion persists in modern definitions of the rodent PFC, frontal cortical areas receive many thalamic inputs beyond the MDT, and the neighboring OFC, premotor, and primary motor cortices also share extensive connections with the MDT [8]. Similarly, conventions that dopaminergic innervation from ventral mesencephalon should define PFC also endure, but the distribution of mesencephalic dopamine projections to the PFC varies across species: more afferents tend to terminate in layers I and III of the ACC and layers V and VI of PL and IL in rats, whereas primates demonstrate greatest distribution in layer I [9, 10].

As no single anatomical criterion successfully delineates the boundaries of the PFC, another approach is to define regions through the determination of the projections and functions of PFC subregions. Ultimately, while rodent PFC is not as differentiated as primate PFC, researchers recognize substantial overlap between rodent mPFC, particularly PL, and primate dorsolateral PFC cognitive and executive functions [11, 12]. Of note, the precentral and ACC areas that were included in early definitions of mPFC are now known to be more involved in sensorimotor processes than the sequencing of actions and outcomes. The ACC is involved in responding to cocaine-associated cues [13] and contexts [14], but overall, this region has been investigated less frequently for cocaine reinstatement than the PL and IL. We also note that, historically, many of the terms used to describe mPFC, including ACC, dorsomedial PFC, and ventromedial PFC, have been conflated [5]. This makes it difficult to discuss mPFC in standardized terms, even within rat studies alone. To that end, we generally defer to the boundaries drawn by Paxinos & Watson, such that PL corresponds with “PrL”, IL with “IL”, and ACC with “Cg1” of the Rat Brain Atlas [15]. We focus on afferent and efferent projections of the mPFC and OFC and their implications for the reinstatement of cocaine seeking.

#### 2.1.1. mPFC afferents

FluoroGold retrograde tracing identified a dorsoventral shift based on mPFC afference: dorsal aspects received predominantly sensorimotor inputs while the ventral aspects received predominantly limbic inputs [16]. The following regions were found to project moderately or heavily to the rodent PL: ACC, IL, insular cortex, medial OFC (mOFC), ventral OFC (vOFC), CA1 and subiculum of hippocampus, claustrum, most midline thalamic nuclei (including MDT, paraventricular, and nucleus reuniens), basolateral amygdala (BLA), basomedial amygdala, and ventral tegmental area (VTA). Conversely, the insular cortex, CA1 and subiculum of the hippocampus, claustrum, most midline thalamic nuclei (including mediodorsal, periventricular, and nucleus reuniens), BLA, basomedial amygdala, and VTA project moderately or heavily to the IL.

While there is much overlap in the regions projecting to both the PL and IL, the strength of innervation differs between these regions. In the hippocampus, ventral CA1 and subiculum project to the ventral mPFC (ventral PL and IL) but not to dorsal mPFC. However, the PL and IL each project to the thalamic nucleus reuniens, which then projects extensively to ventral hippocampus, completing a critical memory loop [17]. Furthermore, a very small population of hippocampal cells that project to PL and IL have collaterals to the nucleus reuniens; a small population of nucleus reuniens neurons that project to hippocampus cells also have collaterals to PL and IL [18].

#### 2.1.2. mPFC efferents

In rodents, the PL sends extensive efferents throughout the brain, including moderate to dense projections to the following regions: IL, ACC, insular cortex, mOFC, medial caudate-putamen, nucleus accumbens core (NAc) and shell (NAs), olfactory tubercle (also known as the tubular striatum; [19]), piriform cortex, BLA, basomedial amygdala, lateral hypothalamus, claustrum, midline thalamic nuclei (including paraventricular and MDT), the VTA, rostromedial tegmentum (RMTg), and the lateral habenula [20–23].

In rodents, the IL projects moderately to densely to: PL, ACC, insular cortex, mOFC, NAc, NAs, piriform cortex, BLA, basomedial amygdala, bed nucleus of stria terminalis, the paraventricular thalamic nucleus, and MDT [21, 22]. Notably, IL terminals are also detected in the lateral and ventrolateral OFC [12].

There is dense interconnectivity of neurons within rodent ACC, PL, and IL. Dorsal mPFC efferents are topographically organized, not simply within a respective cortical layer of mPFC but also across cortical layers with horizontal representation along the medial wall [20]. Additionally, the IL projects densely to ventral PL, hinting at functional relations between ventral mPFC compartments [13, 24].

The PL and IL send projections to common targets, including the mOFC, insular cortex, both the shell and core of the nucleus accumbens (NA), amygdala complex, midline thalamic nuclei, olfactory tubercle, piriform cortex, and one another [17]. Each subregion of the mPFC sends fibers to the NAc and NAs but with slight differences in the strength of the projection: the dorsal PL sends slightly more projections to the NAc than NAs; likewise, the ventral PL sends slightly more efferents to the NAs than NAc [21, 25, 26]. Retrograde tracing using wheat-germ agglutinin with horseradish peroxidase indicates the greatest amount of PL and IL efferents to NAc and BLA emerge from cortical layer V [27]. The same is true for PL and IL efferents to the olfactory tubercle, with the bulk of input arriving from glutamatergic IL neurons [28]. A portion of PL and IL efferents to the amygdala also send collaterals to contralateral mPFC to modulate amygdalar activity across hemispheres [29]. IL- or PL-ipsilateral efferents to the amygdala originate primarily in cortical layers II, III, and V.

The PL and IL, as well as the ACC, send projections to the VTA, arising from cortical layer V [27]. These projections are critical because they influence extracellular dopamine transmission in both the mPFC and NA [30]. Namely, about one-third of such afferents terminate onto VTA dendrites that are reactive for tyrosine hydroxylase, the rate-limiting enzyme for dopamine synthesis. These particular VTA neurons send projections back to mPFC. Another third of mPFC afferents terminate onto VTA neurons that are reactive for GABA and project to the NA [30]. Thus, both PL and IL modulate downstream NA activity through GABAergic means and directly influence the VTA to alter local dopamine release. The VTA also sends substantial GABAergic projections to mPFC [31] but, since these efferents do not receive input from mPFC terminals, they do not form a direct feedback mechanism between the regions [30].

#### 2.1.3. mPFC dorsal-ventral division in connectivity and functionality

In 2003, Heidbreder and Groenewegen [24] published a comprehensive review of mPFC functionality with an emphasis on connectivity, in which they concluded that ventral PL shares more similarities with the IL than dorsal PL. Perhaps most relevant for cocaine reinstatement studies, the ventral PL and IL preferentially project to NAs, whereas the dorsal PL projects largely to the NAc. As a result, ventral PL and IL neurons may be better positioned to mediate VTA dopamine release through NAs intermediary neurons. IL and ventral PL also receive greater projections from CA1, ventral subiculum, and insula than dorsal PL. Heidbreder and Groenewegen further recognized functional similarities between ventral PL and IL. The dorsal PL and ACC are both important for execution of goal-directed behaviors [12]. Meanwhile, the ventral PL and IL are important for development of habitual behavior [32], flexibility in visual/spatial cue discrimination, and integration of physiological states and environmental cues [12]. However, studies using retrograde tracers suggest that there is not a strict PL→NAc and IL→NAs dichotomy. CTb infusions into the NAc label neurons in both the PL and IL [22, 33], with evidence that the IL may send a similar amount of projections to both NAc and NAs [22]. This departs from earlier studies that injected Fluoro-Gold into the medial NAs [10, 34] and the anterograde tracer phaseolus vulgaris leucoagglutinin (PHA-L) into the IL [21].

Heidbreder and Groenewegen’s functional dichotomy was embraced and supported by other researchers [3, 12, 17] and many working with rodent models maintain a dorsal-ventral characterization of the mPFC [3, 5]. While it is largely agreed upon that the ACC is neatly contained in the dorsal mPFC and that the IL is neatly contained within ventral mPFC, there is an ongoing debate as to whether the ventral PL belongs to the dorsal or ventral mPFC.

### 2.2. OFC anatomical boundaries and projections

Historically, subregions of rodent OFC were considered “orbital” areas of the PFC with overlapping functionality. Laubach et al. [5] credit Schoenbaum et al. [35] for functional separation of the OFC from the mPFC based on its role in outcome expectation. Distinct connectional networks between OFC and mPFC have also been identified [36]. Like mPFC, clear boundaries of the OFC were somewhat ill-defined but literature on the OFC suggests less controversy today about how to divide rat OFC subregions than mPFC subregions. The OFC is recognized as encompassing lateral (lOFC), ventrolateral (vlOFC), ventral (vOFC), and mOFC segments based on the presence of dopamine, SMI-32 (a neurofilament protein expressed in pyramidal neurons [37, 38], calbindin, and parval-bumin [39]. OFC subregions can be further divided based on function along a rostral-caudal axis [40]. Based on these criteria, seven distinct categories of OFC function have been proposed: reversal learning, strategy and set-shifting, uncertain outcome encoding, delay discounting, outcome prediction and confidence, reinforcer devaluation, and reward magnitude and identity discrimination. The agranular insular (AI) and dorsolateral cortices are commonly considered OFC subregions in recent literature [40, 41].

Unsurprisingly, there is considerable overlap in afferent and efferent projections of rodent mPFC and OFC. The OFC receives dense projections from regions that process sensory input, including olfactory, auditory, somatosensory, and gustatory regions [42]. The vOFC, vlOFC, and mOFC all receive projections from the posterior parietal and Fr2 motor cortices [43]. vOFC and vlOFC are further innervated by secondary somatosensory areas and occipital zones [43]. The lOFC receives projections from the nearby agranular and granular insular cortices. OFC also receives major projections from regions implicated in reinstatement of cocaine seeking, including thalamus, amygdala, dopaminergic midbrain nuclei, and other cortical regions. All aspects of the submedial thalamic nuclei project to the vlOFC but only the peripheral aspects project to the mOFC, vOFC, and lOFC almost exclusively. All four of the aforementioned OFC subregions receive input from the MDT, which receives direct projections from the NA [44], a major interface between motivation, reinforcement, and drug seeking behavioral output. Yet, most MDT projections target the mOFC, while the more lateral aspects of the OFC are innervated by the BLA and central amygdala [45]. These amygdalar projections mostly terminate in the dorsal AI but some terminate in the dorsal OFC and the ventral AI [46, 47]. The OFC likely integrates processed sensory information and amygdalar output to fulfill its role in decision making. Like other regions of the PFC, including mPFC subregions, the OFC receives dopaminergic projections from midbrain nuclei and the VTA, but only in a small “central zone” extending from lateral aspect of lOFC and into the dorsal AI, and these projections do not collateralize in mPFC [45].

Hoover and Vertes [48] used PHA-L to identify major targets of mOFC and vOFC innervation (confirming results with retrograde FluoroGold tracing). Comparatively, the mOFC distributes projections throughout more of the brain than the vOFC, including to the limbic system, but there is major overlap in their efferent targets. The mOFC sends dense projections to the vOFC, NAc, NAs, PL, dorsal AI, piriform cortex, caudate-putamen, olfactory tubercle, claustrum, hypothalamus, MDT, septum, anterior and central amygdala, periaqueductal gray area, VTA, and the raphe nuclei. With regards to mPFC, the mOFC also sends a moderate amount of projections to the IL. The densest vOFC efferent projections tend to terminate in other regions of cortex, including the dorsal ACC, mOFC, secondary motor, posterior parietal cortex, PL, vOFC, and the temporal association cortices. vOFC also sends dense projections to the caudate-putamen and MDT, which have led some to compare the vOFC with aspects of mPFC [49]. There are relatively few projections to the NA overall [48] and a majority of such projections begin in vOFC or vlOFC and terminate in the NAs [49], some even contralaterally [32]. However, the less dense mOFC→NAc projections should not be over-looked for a possible role in cocaine reinstatement.

In addition to having similarly prominent roles in executive decision making, the mPFC and OFC also share similar cortical, thalamic, and limbic projection networks. Although there is significant overlap, nuanced and behaviorally relevant differences in connectivity do exist. Animal models of cocaine-seeking can help researchers unravel these differences as they pertain to reinstatement.

## 3. Animal models of relapse to cocaine seeking

Animal models of relapse to cocaine seeking allow for the probing of neurocircuits mediating cocaine relapse. Such models aim to simulate acquisition and maintenance of cocaine seeking through operant intravenous self-administration, followed by a drug-free period prior to tests for relapse to drug-seeking. Rodents are commonly used in these models because, like humans, they readily self-administer cocaine. The model of intravenous self-administration involves placing intravenous cocaine delivery under control of an instrumental behavior (e.g., lever press or nose poke). In studies with a focus on the relapse of cocaine-seeking, simple schedules of reinforcement are typically used, such as fixed-ratio 1 (FR-1) schedules in which one lever press or nose poke delivers the drug. However, the effort required to obtain cocaine can be modified through the use of fixed- or progressive-ratio schedules to distinguish between casual and highly motivated cocaine consumption. Cocaine infusions can be paired with discrete visual or auditory stimuli, such as a light and/or tone, to develop an association between the drug and such cues, akin to the human experience.

To investigate relapse to cocaine seeking after a drug-free period, animals are next put through a period of abstinence, during which they typically either remain in the home cage with daily handling (“forced abstinence”), or instrumental extinction training. During instrumental extinction, operant behavior no longer results in presentation of drug-associated cues or drug delivery and responding declines over the course of days to weeks. “Relapse” tests involve a reintroduction to the drug-taking context, the drug itself (usually via a non-contingent priming injection), and/or drug-associated cues. During such tests, if the drug-seeking response is greater than during extinction training, the response is said to have been “reinstated,” and such reinstatement of the drug-seeking response is considered a model of relapse [50]. When animals experience forced abstinence followed by re-exposure to the drug-taking context, during which time the instrumental response once again delivers discrete drug-paired cues (but not the drug itself), animals typically exhibit drug-seeking responses that increase with the length of abstinence [51]. This phenomenon is termed “incubation of cocaine craving” [52], and mirrors the phenomenon observed in some populations of subjects with substance use disorders [53–55].

Rat models of cocaine relapse have been used extensively to identify the neurocircuitry underlying the resumption of cocaine seeking after a drug-free period [56, 57] and are considered to offer reasonable correspondence with relapse and craving in humans [58]. The prefrontal cortex, including OFC, is necessary for cocaine seeking primed by drug-associated cues, stress, and the drug itself [51, 59]. However, defining precise roles of cortical regions in mediating cocaine relapse has been somewhat hindered by differences in anatomical definitions of cortical regions.

## 4. Methods for inhibiting brain regions and circuits

### 4.1. Pharmacological inactivation

In order to determine the necessity of discrete brain regions and circuits for reinstatement of cocaine-seeking, early studies used pharmacological methods to inactivate cells. Several pharmacological tools have been commonly used to inactivate discrete brain regions. Tetrodotoxin (TTX) and lidocaine are highly potent and selective Na^+^ channel blockers that prevent generation of action potentials. Baclofen and muscimol (B+M) are GABA_A_ and GABA_B_ agonists, respectively, often used together to inhibit neuronal activity via presynaptic or postsynaptic hyperpolarization. For example, in the PFC, B+M locally reduces neural activity by inhibiting the presynaptic input onto pyramidal cells, most likely, via activation of presynaptic GABA_B_ receptors [60]. While powerful, there are several inherent disadvantages of pharmacological inactivation studies. First, while the microinfusion of these agents has local and acute effects, this type of intervention lacks temporal and cellular specificity [61]. Indiscriminate inactivation of neurons across an entire region may conceal varying functionalities across neuronal subpopulations within the same region or disrupt proximal unrelated neural pathways. This is especially problematic for a large region involved in many functions, like the mPFC. Conversely, in smaller brain regions, it may be difficult to identify an appropriate dose of drug or rate of infusion to limit the spread of drug. Some degree of tissue damage is unavoidable when infusing pharmacological agents into deeper brain regions using microinjectors. Also, repeated pharmacological inactivation may cause permanent damage, and the precise spread of compounds can be hard to replicate across animals [62]. Additionally, another unintended consequence of lesioning strategies is the recruitment of compensatory mechanisms to offset localized damage. These consequences have encouraged researchers towards more precise cell-type or pathway-specific approaches offered in chemogenetics, optogenetics, and advanced tracing methods.

### 4.2. Chemogenetics

Chemogenetics utilizes genetic technologies (e.g., viral vectors, transgenic animals) to deliver genes encoding “designer receptors” to target cells. Such receptors are termed DREADDs (Designer Receptors Exclusively Activated by Designer Drugs). DREADDs are only activated by an exogenous “designer” ligand, like clozapine N-oxide (CNO, though alternative ligands are utilized as well), after it is administered via systemic injection or microinfusion. Chemogenetic inhibition can be accomplished via AAV-mediated expression of hM4Di receptors, and stimulation via overexpression of hM3Dq receptors, which are both modified muscarinic acetylcholine receptors with high affinity for CNO, and its metabolite clozapine, but not endogenous acetylcholine. Binding at hM4Di receptors results in neuronal inhibition through selective activation of inward-rectifying potassium channels, leading to short-term membrane hyperpolarization [63, 64], while binding at hM3Dq receptors results in depolarization following a G_q_ signaling cascade and release of intracellular Ca^2+^ stores [63, 65]. Chemogenetic strategies are employed because they can be repeated in the same study (namely, within subjects) and offer relatively noninvasive means of exerting timely manipulations in a cell-type or projection- specific manner [64, 66]. Chemogenetics can employ multiple viral vectors to target either pathways or specific subpopulations of cells. Following injection into a brain region, adeno-associated viruses (AAVs) with antero- or retrograde transport capabilities can deposit viral particles encoding DREADDs into presynaptic or postsynaptic neurons relative to the injection site. Yet, pathway-specific retrograde or anterograde approaches alone cannot rule out the effects of collateral targets. Viral vectors can also be employed to express DREADDs in cell subtypes within pathways using viruses with cell-type specific promoters. Additionally, combinatorial viral approaches dependent on Cre-recombination, Tet-Off, or transgenic strategies can be used to target specific pathways or cell-types.

While cell- and circuit-specificity make chemogenetics a tool with significant advantages over pharmacological inactivation, chemogenetic manipulations also have potential off-target effects from the exogenous ligands used to activate the DREADD receptors. For instance, CNO metabolizes into psychoactive compounds clozapine and N-desmethylclozapine in rats and varying doses of CNO can have important behavioral effects: a low dose (1 mg/kg) reduced acoustic startle magnitude and a high dose (5 mg/kg) reduced cocaine-induced hyperlocomotion [67]. Accordingly, the use of appropriate controls to discount off-target effects of compounds like CNO is encouraged. For in-depth reviews of various chemogenetic strategies in behavioral neuroscience, see Gompf et al. [68], Roth [69], Whissell et al. [70], Campbell and Marchant [66], and Smith et al. [64].

### 4.3. Optogenetics

Optogenetics utilizes the expression of photosensitive ion channels, ion pumps, and G-protein coupled receptors (opsins) and allows for temporally precise control of cellular activity and behavior. As with DREADDs, opsins can be targeted to specific projections and cellular populations via the use of transgenic animals, cell-type specific promoters, and combinatorial viral approaches. Three major classes of opsins are employed in optogenetics, and each responds differently to corresponding wavelengths of light: archaerhodopsins, halorhodopsins, and channelrhodopsins. Archaerhodopsins expel H^+^ ions, contributing to membrane hyperpolarization. Similarly, halorhodopsins pump Cl^–^ ions into the membrane and hyperpolarize infected neurons. Channelrhodopsins allow influx of various cations that contribute to depolarization, including Na^+^, K^+^, Ca^2+^, and H^+^ ions. Spatiotemporal control of neuronal membrane potential is a principal advantage of optogenetics [71]. Further, some opsins allow for long-term changes in neural activity following single pulses of light.

As with chemogenetics, optogenetic manipulations can have off-target effects. For instance, one study using optogenetic inactivation in the rat motor cortex reported a larger ensemble, as assayed by extracellular recordings, than what could be confirmed to express the opsin by immunohistochemistry; many of these responses were slow in onset, suggesting that they may have occurred in downstream, uninfected cells through multi-synaptic pathways [72]. It is also imperative to define appropriate stimulation parameters in optogenetic designs as differences in timing and pattern of light stimulation can produce disparate effects [71]. While optogenetics is more temporally and neurophysiologically precise than pharmacologically-based chemogenetics, delivery of light into the brain is invasive and can result in off-target thermal effects if not well-controlled [73]. These challenges affect reproducibility since experimenter validation of actual light power requires careful consideration and calibration [73].

## 5. The role of the PL, IL, and OFC in mediating cocaine reinstatement

### 5.1. PL inactivation reveals a critical role for this brain region in mediating reinstatement

Early studies using pharmacological inactivation approaches have pointed towards a role for the PL in cocaine seeking across various reinstatement models. For example, B+M inactivation of the PL reduced cocaine-primed reinstatement in Sprague Dawley rats when infusions were made exclusively in the PL [74, 75] or at the ACC-PL border [76]. Further, TTX microinfusions into a more ventral location consisting exclusively of the PL of Long Evans rats also reduced cocaine-primed reinstatement, as well as footshock-primed reinstatement [77]. Additionally, TTX-mediated inactivation of both the ACC and PL prevents cue-primed reinstatement in Sprague Dawley rats [78]. And finally, in Wistar rats, lidocaine inactivation of the PL attenuated both cocaine-primed and cue-primed reinstatement when a light+tone complex was used, but not when light+odor complex was used [79]. Taken together, inactivation studies using pharmacological tools provided compelling evidence that rat PL is necessary for cocaine reinstatement primed by discrete cues, cocaine, and footshock.

Bilateral optogenetic inhibition of the PL reduces active lever presses during cocaine-primed reinstatement testing following two weeks of extinction training [80]. However, another study that used a single 90 min extinction session found that PL inhibition reduced cocaine-primed reinstatement only in rats that were allowed to self-administer cocaine at high frequencies, as opposed to low frequency self-administration rats [81]. This indicates that the PL plays an integral role in driving the reinstatement of cocaine seeking in a manner that is influenced by the amount of extinction training received.

#### 5.1.1. Efferent projections from PL: reciprocal PL - BLA projections

Bilateral TTX inactivation of the BLA or dmPFC (specifically PL and ACC) impaired cue-primed cocaine seeking behaviors following extinction [78]. Inactivation of the PL↔BLA circuit via a “disconnection” approach utilizing lidocaine inactivation in contralateral rostral BLA and PL regions inhibited cue-primed reinstatement, while inactivation of ipsilateral BLA and PL alone did not [82]. The same was true for context-primed reinstatement of cocaine seeking after extinction training in a different context [83]. Akin to pharmacological inhibition studies, direct optogenetic inhibition of BLA neurons also reduced cue-primed reinstatement [84]. Interestingly, the same effect was observed when the optogenetic inhibition was limited to a subpopulation of BLA→PL projecting neurons. Although this study did not target specific cell types within PL, it acknowledged that PL targets of BLA afferents include interneurons, which may gate other sensory input to PL in favor of established drug-associated cue information processed by the BLA. Therefore, BLA↔PL pathway inhibition generally attenuates cue-primed reinstatement, but it is not clear whether activation of this pathway will exacerbate cue-primed seeking or which cell types are involved in this modulation. Future optogenetic studies may compare the relative effect of inhibition of BLA afferents in PL GABA interneurons and activation of glutamatergic BLA afferents to PL in cue-primed reinstatement.

#### 5.1.2. Efferent projections from PL: PL to NA

The glutamatergic PL→NAc pathway is critical for cocaine seeking following extinction training. B+M infusions along the ACC-PL border reduce cocaine-primed reinstatement and the NAc glutamate release that drives such reinstatement [76]. These mPFC neurons are dependent on dopamine projections from the VTA to excite their NAc targets [76]. Pharmacological disconnection of the PL→NAc pathway via dopamine receptor antagonism in the PL and glutamate antagonism in the contralateral NAc attenuates cue-primed cocaine-seeking after extinction [22].

Consistent with a role for the PL in cocaine-primed reinstatement, greater PL c-fos expression is evident during cue-primed reinstatement than extinction training [85]. C-fos is an immediate early gene product widely used as a marker for neuronal activation [86]. That study also reported that c-fos expression in PL neurons that project to contralateral NAc positively correlates with active lever presses during cue-primed reinstatement. Similarly, a majority (~75%) of unihemispheric PL→NAc neurons are activated during cue-primed cocaine seeking test after abstinence; however, these neurons make up only about 37% of all c-fos-expressing PL neurons [33]. Approximately 26% of all PL→NAc projections are contralateral [85].

Bilateral optogenetic inhibition of PL→NAc terminals for the duration of testing (2 h) reduced both cocaine- and cocaine+cue-primed reinstatement [80, 87, 88]. This effect may yield synaptic reconfigurations, even after limited optical inhibition periods. Bilateral inhibition of the PL→NAc pathway during the first 15 min of cue-primed reinstatement reduced active lever presses compared to sham protocol animals; yet, both groups exhibited similar cocaine seeking behavior for the remainder of testing when inhibition ceased [89]. Nonetheless, 15 min of laser inhibition within the same study was sufficient to reduce dendritic spine diameter of BLA afferents and AMPA/NMDA ratio within PL. Dense PL innervation also contributes to increased dendritic spine diameter and increased association of perisynaptic astrocytic processes with potentiated spines within NAc [88]. As with optogenetic approaches, DREADD-based inhibition of ACC→NAc and PL→NAc neurons attenuates cocaine- or cue- and cocaine-primed reinstatement, respectively [88, 90].

Cocaine self-administration generates silent synapses in NAc and NAs terminals of mPFC efferent projections and differentially remodels these projections through involuntary abstinence [34]. After 45 days of withdrawal, there was increased surface expression of non-Ca^2+^-permeable AMPA receptors in PL→NAc terminals. The same study also found that optogenetically-induced long-term depression (LTD) of PL→NAc projections reduced cue-primed cocaine seeking after abstinence, suggestive of a pro-seeking function for this pathway.

In summary, pharmacological inactivation and disconnection studies found that the PL→NAc circuit mediates both cue- and cocaine-primed reinstatement. Chemogenetic and optogenetic studies confirm that the PL→NAc pathway is critically important for cue- and cocaine-primed cocaine seeking.

#### 5.1.3. Efferent projections from PL: PL to RMTg

Another PL efferent circuit found to be involved in the reinstatement of cocaine seeking is the PL→RMTg projection. The RMTg receives mostly ipsilateral but some contralateral PL inputs [91]. The RMTg projects to VTA and is implicated in aversive stimulus encoding and inhibition. Functional disconnection of PL (using B+M) and RMTg (using AMPA antagonist NBQX), whether ipsilaterally or contralaterally, enhanced cue-primed reinstatement but not cocaine-primed reinstatement. Optogenetic and chemogenetic strategies have not yet been applied to testing the role of this circuit in mediating the reinstatement of cocaine-seeking. A summary of the methods and findings used in the aforementioned reinstatement or cocaine seeking studies targeting PL is represented in Table 1.

### 5.2. The role of the IL cortex in relapse to cocaine-seeking

The results of pharmacological inactivation studies indicate that the IL inhibits, rather than promotes, reinstatement of cocaine seeking but only after extinction training. For example, B+M infusions into the IL reinstated cocaine seeking (after 11 days of extinction), in the absence of priming by drug-associated cues or drug itself [92]. And further, in the same study, the authors showed that stimulation of the IL via AMPA inhibited cocaine-primed reinstatement. However, the opposite is true in the absence of extinction training. Activation of the IL with bicuculline and baclofen (GABA_A_ and GABA_B_ antagonists) increased cocaine seeking after 1 day of abstinence, while, after 30 days of abstinence, inactivation of IL with B+M attenuated the incubation of cocaine seeking [93]. Similarly, following 21 days of abstinence, intra-IL B+M inhibited cocaine-seeking when the stimulus light over the active (cocaine) lever was used as a discriminative stimulus instead of a response-contingent drug-paired cue [60]. Together, these studies suggest that IL is recruited by extinction training to inhibit cocaine seeking, and in the absence of such instrumental extinction, the IL drives cocaine-seeking.

To the best of our knowledge, only one study to date used an optogenetic or chemogenetic strategy to target the IL during cocaine reinstatement. Optogenetic stimulation of the IL attenuated both cue- and cocaine-primed reinstatement after 21 days of extinction and had no effect on seeking after 21 days of abstinence [94]. This finding potentially indicates that prior work with pharmacological strategies to inhibit the IL following abstinence was able to attenuate incubated cocaine-seeking due to off-target effects in the IL, and specific inhibition of IL neurons does not produce the same effect. Alternatively, it is possible that while IL inhibition can attenuate reinstatement, IL cell body-specific stimulation cannot have the opposite effect due to a ceiling effect. This is unlikely, in light of findings that IL pyramidal neuron activity is necessary for development of enduring extinction of cocaine-seeking [95]. Optogenetic inhibition of IL pyramidal neurons following unreinforced lever presses in extinction training generates resistance to extinction and greater cue-primed reinstatement of cocaine-seeking. The findings suggest IL pyramidal neurons initiate extinction plasticity after lack of reinforcement and this plasticity mediates subsequent reinstatement of cocaine seeking after the presentation of cues but not cocaine. Similar findings were detected using the Daun02 method of ablating activated neuronal ensembles; vmPFC ensembles engaged during instrumental extinction of cocaine-seeking are necessary for subsequent extinction [96]. Thus, it is likely that the IL is critical for extinction learning and subsequent inhibition of cocaine-seeking. However, its role in seeking after abstinence is unclear based on these findings; targeting specific IL efferent pathways may shed light on this issue.

#### 5.2.1. IL in cocaine seeking: efferent projections

Consistent with a role for the IL in the inhibition of reinstatement of extinguished cocaine-seeking, chemogenetic activation of vmPFC neurons projecting to medial NAs attenuates cue-primed, but not cocaine-primed, reinstatement of cocaine seeking [97]. Interestingly, the same effect was not observed when stable step-function opsins were expressed in the IL and optogenetic stimulation occurred via fiber implantation above the NAs [94]. This negative finding may have been due to the fact that these opsins are designed to excite cell bodies, not terminals.

Efforts to delineate a role for IL→NAs pathway in cocaine seeking after abstinence have produced disparate findings. Chemogenetic stimulation of the IL→NAs pathway had no effect on incubated cocaine seeking after 28 days of abstinence [97]. However, the pathway is both temporally and spatially linked to inhibition of drug seeking after home cage abstinence in Long Evans rats [98]. Ca^2+^ imaging indicated that IL→NAs activity decreases shortly before an active lever press, but increased IL→NAs activity afterward is more likely to increase response latency, but not active lever presses, in the next cocaine seeking trial. This is consistent with findings that, whether one day or 30 days into abstinence, IL neurons experience similar phasic changes surrounding operant cocaine seeking behaviors [99]. Following 15 days of abstinence, optogenetic activation of IL→NAs activity during discrete trials in which lever presses deliver cocaine-paired cues, and not in other trials (a within-subjects design), produced a decrease in cue-primed cocaine-seeking during stimulation of the IL→NAs pathway. In another study that used 45 days of abstinence (without extinction training), optogenetically-induced LTD of IL→NAs projections increased active nose pokes, indicative of an antiseeking function for the IL→NAs pathway after abstinence. Therefore, IL→NAs activity is not consistently found to prevent cocaine seeking after abstinence and may depend on stimulation parameters (chemo- vs. optogenetics; specific type of optical stimulation and opsin). Additionally, the Cameron et al. [98] study indicates that changes in lever pressing are not the only indication of IL→NAs activity or plasticity. Many reinstatement studies focus on the number of lever presses or other operant behaviors relative to self-administration or extinction to identify reductions in cocaine seeking behavior. But the temporal specificity of newer techniques, especially optogenetics, permits the analysis of subtle changes in behavior such as latency to engage in cocaine seeking or proximity to cocaine-delivering stimuli. Likewise, measuring responses binned over time or discrete trials may better elucidate how function of IL→NAs pathway changes over the course of drug-seeking or with protracted abstinence, whether in abstinence or extinction paradigms.

## 6. The role of the OFC in reinstatement of cocaine seeking

Early OFC inactivation studies compared effects of excitotoxic lesioning before and after cocaine self-administration training to identify the roles of OFC subregions in cocaine seeking [100]. Lesions were an early strategy employed for targeting the OFC, rather than mPFC, perhaps due to the difficulty in restricting pharmacological agents to and cannulating the smaller mPFC subregions. NMDA-mediated lesions in the mOFC or lOFC did not alter cocaine infusions during self-administration compared to control animals [101]. In fact, the same study found that mOFC- and lOFC-lesioned rats extinguished cocaine seeking behavior and performed similarly to controls during locomotion testing. Findings that OFC-lesioned rats perform similarly to controls is somewhat surprising, given the role of OFC in cue-reward devaluation, set shifting, and cognitive flexibility. This discrepancy may be explained by compensatory action or even the lack of a potent reinforcer to compete against extinction-induced plasticity. The use of FR-1 schedules is also not ideal for comparing reductions in cocaine seeking. When rats undergo a second-order schedule of reinforcement, where cue frequency gradually increases but cocaine was always administered under an FR10 schedule, lesioned rats struggle to advance to higher schedules of cue reinforcement [100]. Thus, the OFC is not categorically implicated in cocaine reinforcement but rather in responding to drug-associated cues and updating their valuation. Accordingly, rats that received pre-training lesions with quinolinic acid also acquired cocaine seeking behaviors sooner, sought more cocaine at lower doses, and responded more on the first day of extinction training than control rats [102]. This readiness to seek cocaine may represent a failure to properly associate cues with the drug experience and, once an association is consolidated, to adjust to new cue values upon a lack of drug availability.

The lOFC is particularly implicated in encoding of drug-associated contexts [103, 104]. Rats were trained to self-administer cocaine and extinguish cocaine seeking in different contexts before undergoing context-primed reinstatement in the self-administration context (ABA design). They received either (1) B+M infusions prior to context-primed reinstatement but after self-administration and extinction training; (2) NMDA-induced lesions before self-administration; or (3) NMDA-induced lesions after self-administration and extinction followed by two additional extinction sessions and then context-primed reinstatement tests. Pre-training NMDA lesions in lOFC increased drug context-primed reinstatement. Again, the lOFC did not appear to inhibit self-administration or extinction training (perhaps due to compensatory mechanisms), but lOFC injury exacerbated context-primed reinstatement behaviors several weeks later.

The lOFC’s specific role in reinstatement is further confirmed by acute pharmacological inactivation. Acute bilateral B+M infusions into lOFC, administered just before reinstatement testing, reduced the cue- [101] and context-primed [103], but not cocaine-primed, reinstatement of cocaine seeking after extinction training. Furthermore, lidocaine inactivation of the lOFC reduced cocaine seeking and flattens cocaine dose-response curves in cocaine-free tests after self-administration training on a second order schedule. Thus, lOFC inactivation may prevent rats from accurately distinguishing between the relative reinforcing properties of the varying cocaine doses [105].

A role for the lOFC and vlOFC in responding to new cue information is closely tied to an interhemispheric relationship with the amygdala. B+M inactivation of the BLA and either ipsilateral or contralateral lOFC attenuated context-primed cocaine seeking after extinction [106]. Notably, compared to control rats, unilateral B+M inactivation of the lOFC failed to attenuate cocaine seeking in the cocaine-paired context. Consequently, one hemisphere of the lOFC must remain intact to process and better respond to changes in cue value.

Similarly implicated in cue-primed reinstatement is the dorsal AI. B+M blockade of the dorsal AI inhibited cue-primed, but not cocaine-primed, reinstatement [41]. This same effect was not observed in food reinstatement, suggesting that the dorsal AI is particularly responsive to cues paired with potent reinforcers. These results are consistent with connectivity between the AI cortex and lOFC [43] and ipsilateral BLA [45].

In human neuroimaging studies, OFC activity diminishes during early withdrawal following a period of initial hypermetabolism [107] but increased cocaine-associated cue reactivity can endure up to 6 months into abstinence [55]. Thus, it is important to measure how cue reactivity and valuation may change in protracted withdrawal in animal models, not just context-primed relapse after extinction training, and the neuroadaptations that underlie those changes. Future inactivation studies may benefit from comparisons of cocaine-induced deficits with lOFC dysfunction [108]. Such designs may offer criterion validity, but alone they fall short of addressing how cocaine self-administration alters the neurobiological substrates within OFC.

Very few studies have employed optogenetics to manipulate OFC activity specifically using models of cocaine seeking. One study found that cocaine self-administration diminished mEPSC frequency in OFC pyramidal neurons [109]. This deficiency was associated with reduced glutamate release in OFC and underlied poor performance in Pavlovian over-expectation tasks. However, performance on these tasks could be corrected using bilateral optogenetic stimulation of the OFC. Whether reduced glutamatergic release is a result of localized or distant afferent dysfunction remains unclear.

The other two optogenetic studies that targeted OFC used an intersectional approach. In the first, a bidirectional optogenetic strategy was used to inhibit either synapses in BLA arising from lOFC, or vice versa, during cue-primed reinstatement [110]. Surprisingly, only inhibition of lOFC efferents in BLA attenuated cue-primed seeking, suggesting that the BLA’s critical role in cue-primed reinstatement is mediated by lOFC. In another surprising finding, optogenetically-induced high-frequency stimulation along the OFC-dorsomedial striatum pathway induces an LTD-like state in striatal targets and reduces animal velocity during locomotion testing [111]. This finding offers another example of cortical inputs depressing striatal targets in cocaine seeking, much like mPFC pyramidal neurons can induce LTD-like states in NAc targets [34]. While limited in number, these optogenetic studies have yielded results that change common understanding of the OFC’s role in cocaine seeking after extinction.

## 7. Conclusions

**Fig. 1.**
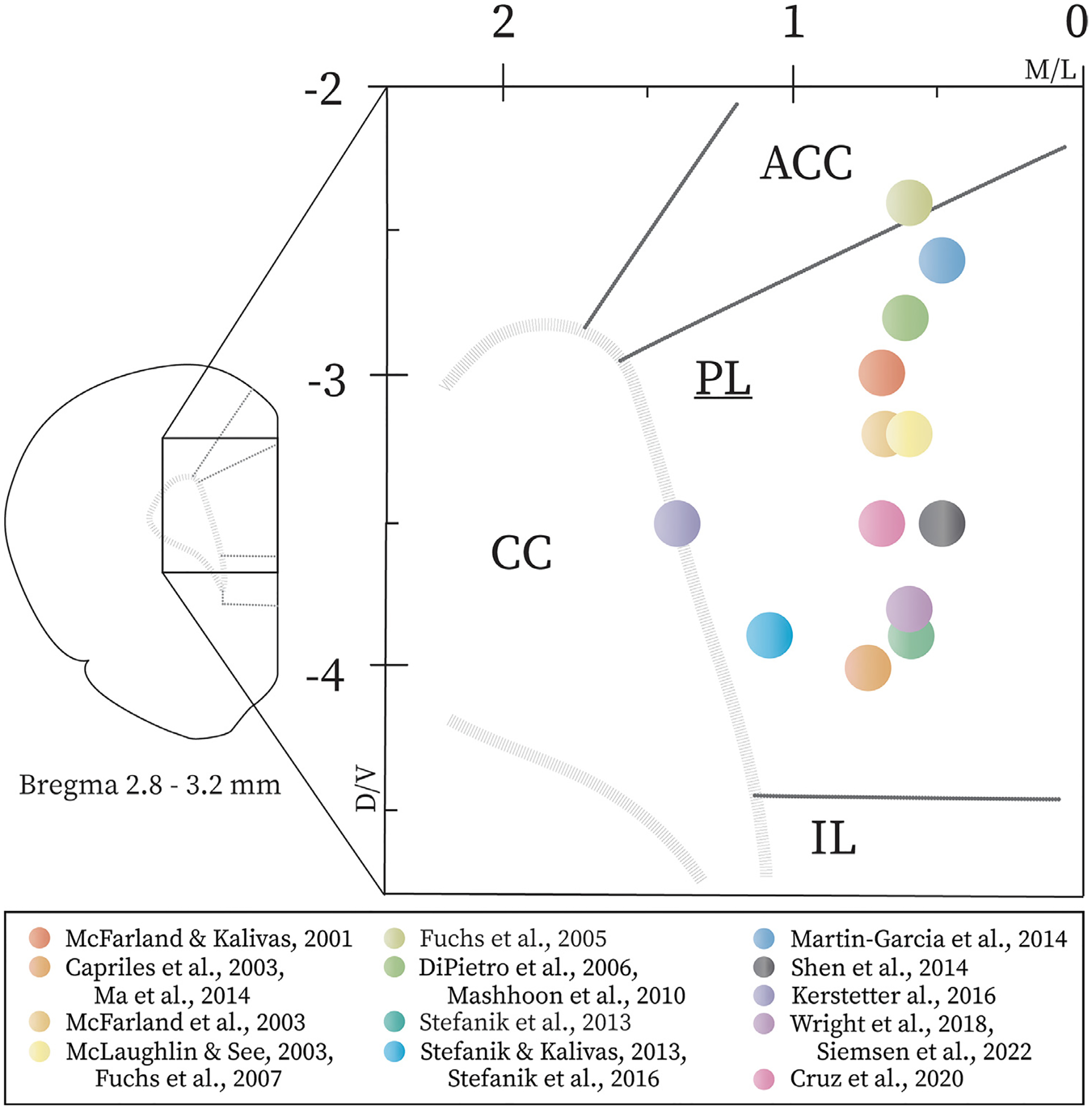
Injection sites of pharmacological and viral compounds within prelimbic cortex used in cocaine reinstatement studies. Targets deduced based on stereotaxic coordinates and microinjection extensions provided in studies and supplemental materials. Studies that used the same targets, based on D/V and M/L dimensions, were combined. Diagram does not account for compound spread. Anterior cingulate cortex = ACC, minor forceps of corpus callosum = CC, infralimbic cortex = IL, prelimbic cortex = PL.

In summary, there is considerable support for the theory that the dmPFC/PL/ACC drives reinstatement of cocaine seeking, and the vmPFC/IL cortex inhibits reinstatement. To date, the precise roles of OFC subregions in cocaine reinstatement remain understudied. Furthermore, the PL drives cocaine seeking, regardless of intracranial injection site, method of inactivation, and reinstatement prime (see Table 1). We assessed the location of the coordinates reported for the injection of pharmacological agents and AAVs into the PL in the studies reviewed here (see Fig. 1). Unsurprisingly, most PL studies seemingly targeted cells within layer V, which maintains most efferents to NA, VTA, and BLA [27, 112]. However, PL injection sites were spread throughout the PL, with no corresponding changes in behavioral outcomes, indicating the PL uniformly guides cocaine seeking. While studies using optogenetic and DREADD approaches have not contradicted the results of earlier work using pharmacological inactivation, chemogenetic and optogenetic studies have permitted the exploration of separate PL pathways. PL→NAc pathway activity underlies both cue- [88, 89] and cocaine-primed reinstatement [80, 87], while the BLA↔PL pathway is evidently critical for cue-primed reinstatement [84]. A role for PL in cocaine seeking exceeds post-extinction reinstatement; recruitment of PL neurons for cocaine seeking is also enhanced after 30 days of abstinence [99]. Recent work by Cruz et al. [91] also implicates the PL→RMTg pathway in cue-primed reinstatement. Despite consistency among most PL findings, there is some evidence that the PL is not limited to pro-seeking functionality and possesses the ability to inhibit cocaine seeking, an ability which may be enhanced by optogenetic stimulation. After eight weeks of self-administration, optogenetic activation of PL enhanced footshock training-induced decreases in cocaine infusions and the latency to seek cocaine in shock-resistant rats [113]. Conversely, optogenetic PL inhibition enhanced cocaine seeking in this model. Moreover, when discriminate stimuli are employed to signal cocaine availability, B+M inhibition of the PL prevents those stimuli from reducing cocaine seeking [114]. B+M inhibition of the PL also prevents the presentation of footshock-associated tones from reducing cocaine seeking [115]. However, reinstatement testing may not be the most effective means of assessing inhibitory control of cocaine seeking, even using advanced methods, considering that prolonged cocaine self-administration and extinction likely alter executive cortical function. Overall, chemogenetic and optogenetic manipulations of the PL corroborate findings from studies employing pharmacological inactivation of the PL, suggestive of agreement across methodologies.

**Fig. 2.**
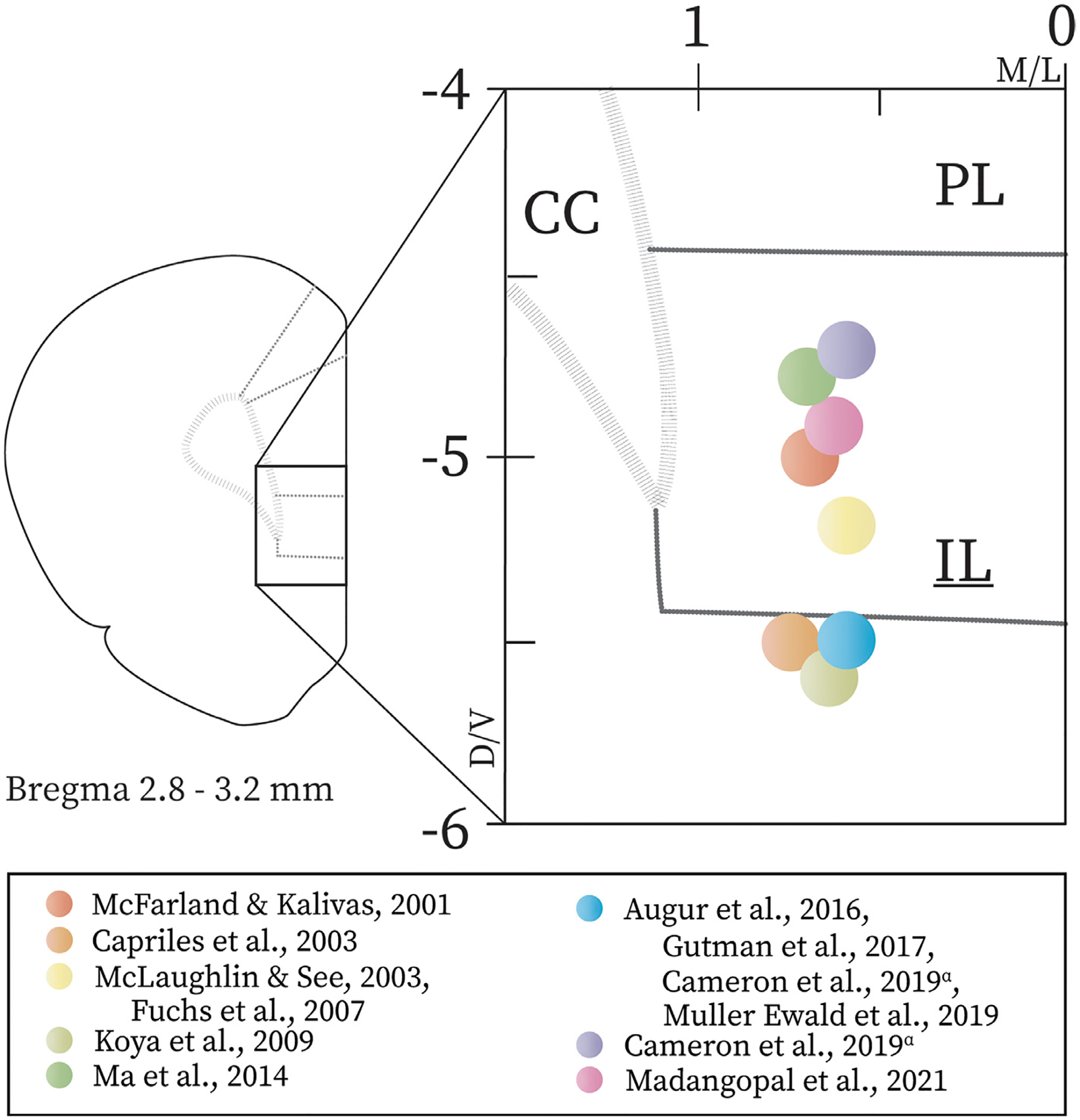
Injection sites of pharmacological and viral compounds within infralimbic cortex used in cocaine reinstatement studies. Targets deduced based on stereotaxic coordinates and microinjection extensions provided in studies and supplemental materials. Studies that used the same targets, based on D/V and M/L dimensions, were combined. Diagram does not account for compound spread. Minor forceps of corpus callosum = CC, infralimbic cortex = IL, prelimbic cortex = PL ^*α*^ Study involved viral injection at two separate D/V coordinates: −4.7 and −5.5 mm.

Our overview of the literature on the role of the IL in reinstatement provides a less cohesive story than currently exists for the PL. Consistent with pharmacological inactivation studies, studies using chemogenetics and optogenetics indicate that extinction training recruits the IL to inhibit cocaine seeking, regardless of varying target coordinates within IL (see Fig. 2 and Table 2). However, in the absence of extinction training, pharmacological inactivation of the vmPFC attenuates incubated cocaine seeking [93], while chemogenetic stimulation has no effect [34, 94, 97, 98]. The vmPFC as targeted by Koya et al. was slightly more ventral than other stereotaxic IL targets used by other studies reviewed (Fig. 2). This raises the possibility that the pharmacological inactivation employed by Koya et al. [93] had off-target effects or manipulated ensembles that were not recruited against cocaine seeking by extinction. While a number of studies have *stimulated* the vmPFC itself or its efferents, assessing the consequences of inhibiting this region via chemo- or optogenetics is necessary to provide clarity on the role of this brain region after abstinence without extinction. Optogenetic inhibition of IL pyramidal neurons prevents the consolidation of extinction [95]. Optogenetic activation of IL decreases cocaine seeking in both cue- and cocaine-primed seeking tests, but only after extinction [94]. In the absence of extinction training, the role of the IL and its efferents may change with time, perhaps becoming more implicated in cocaine seeking rather than cocaine-associated cue devaluation ([93, 94] regarding IL→NAs connection). When interpreting results of reinstatement studies, it should also be considered that both non-contingent cocaine- and cue-primed reinstatement tests are themselves extinction sessions; throughout the test periods, the lack of drug yields following operant responding likely promotes severance of the response-cocaine relation. The IL may be critical for updating of responses to cocaine-associated cues [116] and, if so, future work should seek to distinguish the respective roles of the IL, mOFC, and lOFC in cocaine-associated cue revaluation [101]. The IL projection network includes regions implicated in cue-primed reinstatement, including afferents from BLA [16] and reciprocal projections with the mOFC [21, 48]. Chemogenetic or optogenetic manipulation of BLA↔IL and IL↔mOFC pathways during extinction and cue-primed reinstatement might help discern whether communication across the IL projection network, as opposed to the IL alone, is necessary for cocaine-associated cue revaluation.

While IL→NAs pathway has rightfully been the focus of several reinstatement studies [92, 94, 97], CTb retrotracing suggests there may be no significant differences between the number of IL→NAs and IL→NAc projections [22], although other tracing techniques might provide additional, more granular information. In agreement with the idea that the IL projections to the NAc are significant in number and involved in cocaine relapse, the number of c-fos+ cells co-labeled with CTb were similar for both PL and IL when CTb was delivered to the NAc and a cued relapse test was conducted after 14 days of abstinence from cocaine self-administration [33]. Although evidence suggests that IL→NAc pathway contains self-administration ensembles [96], researchers have yet to identify a clear role for IL→NAc neurons in reinstatement. Future studies should also employ chemogenetic or optogenetic manipulations to inhibit IL→NAc pathway during extinction and cue-primed reinstatement because it is possible that such designs may address whether the pathway is responsible for IL’s pro-seeking functionality. Another approach to determine the IL’s multi-faceted role in reinstatement should emphasize neuronal ensembles within IL, as opposed to viewing IL function as monolithic. Different ensembles are implicated in self-administration than extinction [96]. Likewise, 30 days into abstinence from self-administration, IL neuronal activity precedes and follows cocaine seeking operant behaviors during extinction tests and renewed self-administration [99]. Interestingly, different populations of cells were implicated in these different phases of testing. Looking beyond the simplistic understanding of IL-NAs projection seems critical for deciphering ongoing uncertainty about the role of the IL in reinstatement.

Regarding OFC, there continues to be limited data on its role in cocaine reinstatement. Namely, bidirectional optogenetics confirms a critical role for lOFC→BLA pathway in cue-primed cocaine seeking [110]. Beyond the Arguello study, however, we conclude that the role of OFC in cocaine reinstatement, especially cue-primed reinstatement, remains understudied. There is, however, considerable evidence of OFC dysfunction in humans after cocaine cessation [107] and in rodents after cocaine self-administration [117], suggesting a need to target the OFC in reinstatement studies. The mOFC→NAc pathway is associated with impulsivity in reward seeking when using a water reward [47]. mOFC→NAc pathway may experience more profound changes after chronic exposure to a potent reinforcer like cocaine, which demonstrably reduces OFC activity in human users. Activation of this pathway during extinction or the latter periods of a cue-primed reinstatement test may likewise reduce cue-primed drug seeking. The vOFC is potentially a key region in the revaluation of cocaine-associated cues based on similar c-fos expression as the lOFC during cue-primed reinstatement [118], its role in reversal learning and set shifting, and dense projections to the IL. Likewise, the dorsal AI is implicated in cue-primed reinstatement [41] and receives projections from the BLA [46]. Moreover, it is possible that both the IL and OFC are implicated in revaluation of drug-associated cues and contexts. Accordingly, future reinstatement studies should seek to disentangle their respective roles in reinstatement (perhaps by manipulating connectivity between the IL and neighboring mOFC), which could also have greater implications for other models of associative learning.

As a final reminder, all studies reviewed here (see Tables 1 and 2) used male rats exclusively. Sexually dimorphic PFC activation following cocaine use has been measured in both rodents [13] and humans [119]. The importance of understanding sex differences in reinstatement studies cannot be overstated and future studies should compare results across sexes.

Chemogenetics and optogenetics each permit interrogation of PFC pathways with temporal and cell-type specificity unavailable by means of pharmacology alone. Even so, nearly all studies using these methods have largely corroborated prior pharmacologically-based findings about roles of the PL and IL in reinstatement. These methods should also improve future understanding of OFC function in cue-primed reinstatement. We especially encourage the use of combinatorial approaches when appropriate to specifically identify the roles of neuronal subpopulations in a given region or pathway of interest. Understanding the roles of each pathway may help unravel the complex webbing of cocaine seeking behaviors and identify potential targets for pharmacological intervention.

## Figures and Tables

**Table 1:** Role of PL cortex in cocaine reinstatement and incubation of craving studies targeting PL cortex.

	REFERENCE	STRAIN	REGION(S) OF PL TARGETS	PL TARGETS	METHOD	ACTIVATION OR INHIBITION	BEHAVIORAL RESULTS
PL OR DMPFC REGION	McFarland & Kalivas, 2001	Sprague Dawley	Bilateral dorsal PFC	AP +3.0 mm, ML ±0.7 mm, DV −3.0 mm	Pharmacological: baclofen + muscimol	Inhibition	Regional dorsal PFC inhibition decreased cocaine-primed reinstatement
	Capriles et al., 2003	Long Evans	Bilateral PL	AP +3.2 mm, ML ±0.75 mm, DV −4.0mm	Pharmacological: tetrodotoxin	Inhibition	Regional sodium blockade in PL decreased both footshock- and cocaine-primed reinstatement
	McFarland et al., 2003	Sprague Dawley	Bilateral dorsal PFC	AP +3.0 mm, ML ±0.7 mm, DV −3.2mm	Pharmacological: baclofen & muscimol in PL-ACC border	Inhibition	Regional dorsal PFC inhibition decreased cocaine-primed reinstatement
	McLaughlin & See, 2003	Sprague Dawley	Bilateral PL	AP +3.2 mm, ML ±0.6 mm, DV −3.2mm	Pharmacological: tetrodotoxin	Inhibition	Regional PL inhibition decreased cue-primed reinstatement
	Fuchs et al., 2005 ^α^	Sprague Dawley	Bilateral dmPFC	AP +3.0 mm, ML ±0.6 mm, DV −2.4mm	Pharmacological: tetrodotoxin in ventral PL and IL	Inhibition	Regional PL and IL inhibition did not affect context-primed reinstatement
	Di Pietro et al., 2006	Wistar	Bilateral PL	AP +2.8 mm, ML ±2.0 mm, DV −4.5 mm (at 30° angle) OR AP +2.8 mm, ML ±1.4 mm, DV −3.9 mm (at 15° angle)	Pharmacological: lidocaine	Inhibition	Regional PL sodium blockade decreased (light+ tone) cue- and cue+cocaine-primed reinstatement, but not light+odor cue-primed reinstatement
	Stefanik et al., 2013	Sprague Dawley	Bilateral PL	AP +3.1 mm, ML ±0.6 mm, DV −3.9mm	Optogenetic: rAAV2-hSyn-eNpHR3.0-EYFP	Inhibition	Optogenetic inhibition of PL pyramidal neurons decreased cocaine-primed reinstatement
	Martín-García et al., 2014	Sprague Dawley	Bilateral PL	AP +3.2 mm, ML ±0.5 mm, DV −2.6mm	Optogenetic: AAV-CamKII-ArchT-EYFP Optogenetic: AAV-CamKII-hCHR2(H134R)-EYFP	Inhibition Activation	Following high frequency cocaine intake, inhibition of excitatory PL neurons decreased cocaine-primed reinstatement Following low frequency cocaine intake, activation of excitatory PL neurons did not significantly affect cocaine-primed reinstatement (did not differ from EYFP controls)
	Shen et al., 2014	Sprague Dawley	Bilateral PL	AP +3.2 mm, ML ±0.5 mm, DV −3.5mm	Pharmacological: baclofen + muscimol	Inhibition	Regional PL inhibition decreased cocaine-primed reinstatement
PL- OR DMPFC-BLA PATHWAY	Fuchs et al., 2007	Sprague Dawley	Unilateral dmPFC	AP +3.0 mm, ML ±0.6 mm, DV −3.2mm	Pharmacological disconnection: baclofen + muscimol in unilateral BLA and either ipsi- or contralateral PL	Inhibition	Both ipsilateral and contralateral PL↔BLA disconnection approaches decreased reinstatement induced by the cocaine-paired context, but not the extinction context.
	Mashhoon et al., 2010	Wistar	Unilateral PL	AP +2.8 mm, ML ±1.4 mm, DV −3.9 mm (at 15° angle)	Pharmacological disconnection: lidocaine in unilateral BLA and contralateral PL	Inhibition	Contralateral PL↔BLA sodium blockade decreased cue-primed reinstatement under second order schedule, but exclusive regional PL or BLA sodium blockade did not (design did not test ipsilateral PL-BLA disconnection)
	Stefanik & Kalivas, 2013	Sprague Dawley	BLA terminals in PL	AP +3.1 mm, ML +2.0 mm, DV −4.0 mm (at 12° angle)	Optogenetic: rAAV2-CAG-ArchT-GFP	Inhibition	Optogenetic inhibition of BLA terminals in PL decreased cue-primed reinstatement
PL- OR DMPFC–NAC PATHWAY	Stefanik et al., 2013	Sprague Dawley	PL terminals in NAc	AP +3.1 mm, ML ±0.6 mm, DV −3.9mm	Optogenetic: rAAV2-CAG-ArchT-GFP	Inhibition	Optogenetic inhibition of PL terminals in NAc decreased both cocaine-primed and cue+cocaine primed reinstatement
	Ma et al., 2014	Sprague Dawley	Bilateral PL→NAc pathway	AP +3.0 mm, ML ±0.75 mm, DV −4.0mm	Optogenetic: AAV2-hCHR2(H134R)-EYFP	Activation	After 45 days of withdrawal, optogenetically-induced long-term depression of PL→NAc pathway decreased cue-induced cocaine seeking during extinction test
	Kerstetter et al., 2016	Long Evans	Bilateral mPFC→NAc pathway	AP +3.2 mm, ML ±1.4 mm, DV −3.5mm	Chemogenetic: AAV-hSYn-DIO-hM_4_ Di-m-Cherry	Inhibition	Chemogenetic inhibition of PL→NAc pathway decreased cocaine-primed reinstatement
	Stefanik et al., 2016	Sprague Dawley	PL terminals in NAc	AP +3.1 mm, ML ±2.0 mm, DV −4.0 mm (at 12° angle)	Optogenetic: rAAV2-CAG-ArchT-GFP	Inhibition	Optogenetic inhibition of PL terminals in NAc decreased cue-primed reinstatement test; effect did not persist without laser stimulation.
	Wright et al., 2018	Sprague Dawley	mPFC terminals in NAc	AP +3.1 mm, ML ±0.6 mm, DV −3.9mm	Optogenetic: AAV-CamKII*α*-eNpHR3.0-EYFP	Inhibition	Optogenetic inhibition of PL terminals in NAc decreased cocaine-primed reinstatement
	Siemsen et al., 2022	Sprague Dawley	Bilateral PL→NAc pathway Bilateral PL→NAc pathway	AP +2.8 mm ML ±0.6 mm DV −3.8 mm AP +2.8 mm ML ±0.6 mm DV −3.8 mm	Chemogenetic: AAV2-hSYn-DIO-hM4Di OR Chemogenetic: AAV1-CaMKII*α*-Cre (transsynaptic)	Inhibition Inhibition	Chemogenetic inhibition of PL→NAc pathway decreased cue-primed reinstatement across time bins (after a period of abstinence followed by extinction) Chemogenetic inhibition of most dense and active PL→NAc neuron subpopulation decreased cue-primed reinstatement of cocaine seeking, but not sucrose seeking
PL-RMTG PATHWAY	Cruz et al., 2020	Sprague Dawley	PL alone PL→RMTg pathway	AP +3.1 mm, ML ±1.5 mm, DV −3.5–3.7 mm (at 12° angle)	Pharmacological: baclofen + muscimol Pharmacological disconnection: baclofen + muscimol in unilateral PL and AMPA antagonist NBQX in either ipsi- or contralateral rostromedial tegmentum	Inhibition Inhibition	Unilateral PL inhibition did not affect cue-primed reinstatement Both ipsilateral and contralateral PL→RMTg disconnection approaches increased cue-primed reinstatement. Contralateral PL→RMTg disconnection did not affect cocaine-priemd reinstatement (did not assess ipsilateral PL→RMTg pathway in cocaine-primed reinstatement)

All stereotaxic coordinates are relative to bregma. DV coordinates account for end of surgically inserted guide cannulae and extension by internal infusion cannulae or optic probes.

α This study aimed to pharmacologically inactivate “dmPFC” but published figures indicate injection sites were almost exclusively in what was considered ACC, not PL.

**Table 2:** Role of IL cortex in cocaine reinstatement and incubation of craving studies targeting IL cortex.

	REFERENCE	STRAIN	REGION(S) OF INTEREST	IL TARGETS	METHOD	ACTIVATION OR INHIBITION	BEHAVIORAL RESULTS
IL OR VMPFC REGION	McFarland & Kalivas, 2001	Sprague Dawley	Bilateral ventral PFC	AP +3.0 mm, ML ±0.7 mm, DV −5.0 mm	Pharmacological: baclofen + muscimol	Inhibition	Regional ventral PFC inhibition did not affect cocaine-primed reinstatement
	Capriles et al., 2003	Long Evans	Bilateral IL	AP +3.2 mm, ML ±0.75 mm, DV −5.5mm	Pharmacological: tetrodotoxin	Inhibition	Regional sodium blockade in IL did not affect reinstatement induced by footshock or non-contingent cocaine injection
	McLaughlin & See, 2003	Sprague Dawley	Bilateral IL	AP +3.2 mm, ML ±0.6 mm, DV −5.2mm	Pharmacological: tetrodotoxin	Inhibition	Regional IL inhibition decreased cue-primed reinstatement
	Fuchs et al., 2005	Sprague Dawley	Bilateral vmPFC	AP +3.0 mm, ML ±0.6 mm, DV −5.2mm	Pharmacological: tetrodotoxin in ventral PL and IL	Inhibition	Regional ventral PL and IL inhibition did not affect context-primed reinstatement
	Peters et al., 2008	Sprague Dawley	Bilateral IL	Not specified; based on Paxinos & Watson (1986), AP +3.0 to 3.7 mm for IL	Pharmacological: baclofen + muscimol Pharmacological: AMPA	Inhibition Activation	Regional IL inhibition increased cocaine-primed reinstatement Regional IL activation decreased cocaine-primed reinstatement
	Koya et al., 2009	Long Evans	Bilateral vmPFC	AP +2.8 mm, ML ±0.6 mm, DV −5.6mm	Pharmacological: baclofen + muscimol Pharmacological: bicuculline + saclofen	Inhibition Activation	vmPFC inhibition decreased cue-induced cocaine seeking after 30 days, but not 1 day, of withdrawal vmPFC activation increased cocaine seeking after 1 day, but not 30 days, of withdrawal
	Gutman et al., 2017	Sprague Dawley	Bilateral IL	AP +3.0 mm, ML ±0.6 mm, DV −5.5mm	Optogenetic: AAV5-CaMKII*α*-eArchT3.0-eYFP	Inhibition	IL pyramidal neuron inhibition (following unreinforced lever pressing during extinction) increased cocaine seeking during extinction and subsequent cue-primed reinstatement but not during cocaine-primed reinstatement
	Muller Ewald et al., 2019	Sprague Dawley	Bilateral IL	AP +2.8 mm, ML ±0.6 mm, DV −5.5 mm	Optogenetic: AAV5-CaMKII*α*-hChR2(C128S/D156A)-eYFP, a stable-step function opsin	Activation (SSFO-based)	Optogenetic IL activation decreased both cue-and cocaine-primed reinstatement but only after extinction training
	Madangopal et al., 2021	Sprague Dawley	Bilateral IL	AP +3.0 mm, ML ±1.5 mm, DV −5.0 mm (at 10° angle)	Pharmacological: baclofen + muscimol	Inhibition	Regional IL inhibition decreased cocaine seeking during both DS+ and DS− trials
IL- OR VMPFC–NAS PATHWAY	Peters et al., 2008	Sprague Dawley	Unilateral IL	Not specified; based on Paxinos & Watson (1986), AP +3.0 to 3.7 mm for IL, AP 1.1 to 2 mm for NAs	Pharmacological disconnection: baclofen + muscimol in ipsi- or contralateral IL-NAs pathway	Inhibition	Both ipsi- or contralateral IL-NAs disconnection approaches increased cocaine-primed reinstatement
	Ma et al., 2014	Sprague Dawley	Bilateral IL→NAs pathway	AP +3.0 mm, ML ±0.70 mm, DV −4.75mm	Optogenetic: AAV2-hCHR2(H134R)-EYFP	Activation	After 45 days of withdrawal, optogenetically-induced long-term depression of IL-NAs pathway increased cue-induced cocaine seeking during extinction test
	Augur et al., 2016	Sprague Dawley	Bilateral vmPFC; vmPFC→NAs pathway	AP +2.9 mm, ML ±0.6 mm, DV −5.5 mm	Chemogenetic: Cre-dependent AAV2-hSyn-DIO-hM3D(Gq)-mCherry	Activation	Chemogenetic activation of vmPFC–NAs pathway reduced cocaine seeking during cue-primed reinstatement
	Cameron et al., 2019	Long Evans	Bilateral IL→NAc pathway	AP +3.1 mm, ML ±0.6 mm, DV −4.7 and −5.5mm	Optogenetic: AAV2/5-CamKII-hChR2(H134R)-eYFP	Activation	Optogenetic activation of NAs afferent neurons in IL (1) reduced cocaine seeking after days 1 and 15 of abstinence and increased latency to respond during the subsequent trial
	Muller Ewald et al., 2019	Sprague Dawley	IL terminals in NAs	AP +2.8 mm, ML ±0.6 mm, DV −5.5 mm	Optogenetic: AAV5-CaMKII*α*-hChR2(C128S/D156A)-eYFP, a stable-step function opsin	Activation (SSFO-based)	Optogenetic activation of IL terminals in NAs did not significantly affect cocaine seeking during reinstatement

All stereotaxic coordinates are relative to bregma. DV coordinates account for end of surgically inserted guide cannulae and extension by internal infusion cannulae or optic probes.
